# Intranasal MMI-0100 Attenuates Aβ_1−42_- and LPS-Induced Neuroinflammation and Memory Impairments via the MK2 Signaling Pathway

**DOI:** 10.3389/fimmu.2019.02707

**Published:** 2019-11-26

**Authors:** JinHong Jiang, Zhe Wang, XueYa Liang, YaoYan Nie, Xin Chang, HongXiang Xue, Shu Li, Chang Min

**Affiliations:** ^1^Institute of Biochemistry and Molecular Biology, School of Life Sciences, Lanzhou University, Lanzhou, China; ^2^Jiangsu Province Key Laboratory in Anesthesiology, School of Anesthesiology, Xuzhou Medical University, Xuzhou, China; ^3^School of Basic Medical Sciences, Xi'an Jiaotong University, Xi'an, China

**Keywords:** MMI-0100, neuroinflammation, MAPK-activated protein kinase II (MK2), intranasal, near-infrared fluorescent

## Abstract

**Background:** Accumulating evidence suggests inhibiting neuroinflammation as a potential target in therapeutic or preventive strategies for Alzheimer's disease (AD). MAPK-activated protein kinase II (MK2), downstream kinase of p38 mitogen activated protein kinase (MAPK) p38 MAPK, was unveiled as a promising option for the treatment of AD. Increasing evidence points at MK2 as involved in neuroinflammatory responses. MMI-0100, a cell-penetrating peptide inhibitor of MK2, exhibits anti-inflammatory effects and is in current clinical trials for the treatment of pulmonary fibrosis. Therefore, it is important to understand the actions of MMI-0100 in neuroinflammation.

**Methods:** The mouse memory function was evaluated using novel object recognition (NOR) and object location recognition (OLR) tasks. Brain hippocampus tissue samples were analyzed by quantitative PCR, Western blotting, and immunostaining. Near-infrared fluorescent and confocal microscopy experiments were used to detect the brain uptake and distribution after intranasal MMI-0100 application.

**Results:** Central MMI-0100 was able to ameliorate the memory deficit induced by Aβ_1−42_ or LPS in novel object and location memory tasks. MMI-0100 suppressed LPS-induced activation of astrocytes and microglia, and dramatically decreased a series of pro-inflammatory cytokines such as TNF-α, IL-6, IL-1β, COX-2, and iNOS via inhibiting phosphorylation of MK2, but not ERK, JNK, and p38 *in vivo* and *in vitro*. Importantly, one of the reasons for the failure of macromolecular protein or peptide drugs in the treatment of AD is that they cannot cross the blood–brain barrier. Our data showed that intranasal administration of MMI-0100 significantly ameliorates the memory deficit induced by Aβ_1−42_ or LPS. Near-infrared fluorescent and confocal microscopy experiment results showed that a strong fluorescent signal, coming from mouse brains, was observed at 2 h after nasal applications of Cy7.5-MMI-0100. However, brains from control mice treated with saline or Cy7.5 alone displayed no significant signal.

**Conclusions:** MMI-0100 attenuates Aβ_1−42_- and LPS-induced neuroinflammation and memory impairments via the MK2 signaling pathway. Meanwhile, these data suggest that the MMI-0100/MK2 system may provide a new potential target for treatment of AD.

## Highlights

- Central MMI-0100 is able to ameliorate Aβ_1−42_- or LPS-induced memory deficit.
- MMI-0100 suppresses LPS-induced activation of astrocytes and microglia *in vivo*.
- MMI-0100 inhibits inflammatory factors via MK2 pathway *in vivo* and *in vitro*.
- Intranasal infusion of MMI-0100 ameliorates Aβ_1−42_- or LPS-induced memory deficit.
- The brain uptake of Cy7.5-MMI-0100 was detected by near-infrared fluorescent.

## Introduction

Alzheimer's disease (AD), one of the most common neurodegenerative diseases, is characterized by progressive cognitive dysfunction, memory impairment, and behavioral changes. The pathological hallmarks of AD are amyloid-β (Aβ) plaques and neurofibrillary tangles, accompanied by neuroinflammation characterized by the activation of microglia, and astrocytes (1–6). Increasing evidences suggest that microglia, the first line of defense and the resident macrophages of the brain in the central nervous system (CNS), play multiple roles in AD progression by clearing Aβ plaques and releasing pro-inflammatory mediators [e.g., tumor necrosis factor-α (TNF-α), interleukin-6 (IL-6), and interleukin-1β (IL-1β)], which might directly act on neurons to induce apoptosis (1, 4, 6, 7). AD pathogenesis is not restricted to the neuronal compartment, but includes strong interactions with immunological mechanisms in the brain. Targeting of these immune mechanisms could lead to future therapeutic or preventive strategies for AD. An abundance of clinical trials evaluating anti-inflammatory drugs in AD or mild AD is underway, including NCT02423200, NCT02423122, NCT01009359, NCT02062099, NCT00013650, and NCT02646982.

The p38 mitogen-activated protein kinase (MAPK) signaling pathway, a cascade contributing to neuroinflammation, could play a key role in AD pathophysiology, and preclinical and clinical trials have evaluated the pharmacological effects of p38 MAPKα inhibitors in the brain (8, 9). Bachstetter et al. demonstrated that the effects of the p38 MAPK inhibitor MW01-2-069A-SRM in neuroinflammatory induced by toll-like receptor (TLR) ligands or Aβ is associated with decreased phosphorylation state of mitogen-activated protein kinase activated protein kinase II (MK2) (10). Culbert et al. point at elevated activation and expression of MK2 correlated with Aβ deposition, microglial activation, and pro-inflammatory mediator upregulation in the transgenic mouse model of Alzheimer's disease (AD) (11). MK2 is downstream kinase of p38 MAPK, and suppression of MK2 activity also leads to down-regulation of inflammatory cytokine expression such as TNF-α, IL-1β, and IL-6. Due to side effects of p38 inhibitors in hepatotoxicity, cardiotoxicity, and undisclosed CNS toxicity in clinical trials, it is an unacceptable safety profile for drug development (12–15). Thus, MK2 was unveiled as a promising option for the treatment of neural diseases.

MMI-0100, a recently discovered cell-permeable peptide inhibitor of MK2, consists of 22 amino acids in the sequence YARAAARQARAKALARQLGVAA. YARAAARQARA is a cell-permeable peptide (CPP) named PDT-4, and it provided more specificity to inhibit MK2 (16). Phase 1a study investigated the effect of MMI-0100 on airway inflammation with inhaled lipopolysaccharide (LPS) and currently in clinical trials for the treatment of pulmonary fibrosis (NCT02515396) (17). Therefore, we are eager to investigate whether MMI-0100 suppress Aβ- and LPS-induced neuroinflammation and attenuates memory impairment.

A major challenge of protein and peptide drugs for the treatment of neurodegenerative diseases is the delivery of these drugs over the blood–brain barrier (BBB). Solanezumab's failure in the Phase III trial inspired us to determine whether brain-targeted drugs can cross the BBB (mainly in any dose of antibody, and ultimately only about 0.1% can penetrate the BBB to reach the brain) (18). Intranasal (IN) infusion allows drugs to rapidly enter the CNS via extracellular trigeminal and intracellular neuronal olfactory associated pathways, bypassing the BBB to effectively interact 0 with multiple brain regions (19–22). Given that MMI-0100 is a cell-permeant MK2 inhibitor peptide, we also evaluated whether intranasal administration of MMI-0100 could regulate Aβ- and LPS-induced neuroinflammation.

## Materials and Methods

### Animals

Male Kunming mice, a Swiss strain, 8–10 weeks old, weighing 20–24 g, were obtained from the Experimental Animal Center of Lanzhou University (Lanzhou, China). All mice were housed in 20 × 30 cm^2^ cages (humidity 45–50%, sizes 20 × 30 cm^2^, bedding-wood shavings, 5 animals/cage) with free access to tap water and food in a room. The temperature of the room was maintained at 22 ± 2°C and was accompanied by a 12-h light–dark cycle (8:00 a.m.−8:00 p.m.). All the protocols in our experiments were approved by the Ethics Committee of Lanzhou University (permit number: SYXK Gan 2009–0005).

### Surgery

An 8-mm 26-gauge stainless steel guide cannula was implanted in the lateral ventricle or bilateral hippocampus according to our previous reports (23, 24). Each mouse was anesthetized with sodium pentobarbital at a dose of 70 mg/kg (Sigma, USA) and fixed in a stereotaxic frame (Leica, Germany). According to the atlas of Paxinos and Franklin (25), guide cannulas were implanted over the lateral ventricle (AP = 0.5 mm, RL = 1.0 mm, DV = 2.0 mm) or were implanted in the bilateral hippocampus (AP = 2 mm, RL = ±1.5 mm, DV = 1.2 mm). Each mouse was housed individually and allowed to recover for 5–7 days after surgery. All experiments were conducted between 9:00 a.m. and 6:00 p.m.

At the end of the experiments, each mouse was sacrificed and brains were then dissected, and the position of each cannula was determined by injecting methylene blue into the cannula. Data exhibiting the diffusion of methylene blue in the ventricles were analyzed in the statistical evaluation. In addition, the bilateral hippocampus position was checked by hematoxylin–eosin (H&E) staining (23). All mice were used only once.

### Drug Application

MMI-0100 (YARAAARQARAKALARQLGVAA) was synthesized by a standard Fmoc-based solid-phase synthetic method, and refer to our previous reports for detailed methods (23). Purified MMI-0100 was dissolved in saline (vehicle) at a concentration of 10 nmol/μl, stored at −20°C after reconstitution, and diluted to 2.5 nmol/μl in saline immediately before being injected. Stability of MMI-0100 in mouse brain homogenates was investigated and its half-life is about 161.7 minutes. The detail results are shown in Figure S5. Aβ_1−42_ peptide was purchased from Hangzhou Chinese Peptide Biochemical Company. Aβ_1−42_ was first dissolved in hexafluoroisopropanol and frozen at −80°C, and it was directly lyophilized to form a powder, which is Aβ_1−42_ oligomer. Aβ_1−42_ was dissolved in 5% DMSO in saline, kept at −20°C, and was diluted in saline immediately before injected (800 pmol/mouse). Chronic injection of the Aβ_1−42_ oligomer at 14 days prior to the start of the experiment is a widely used AD model (26–29). LPS was bought from Sigma (Sigma-Aldrich Co., USA). LPS (2 μg/mouse) was dissolved in saline and infused into the lateral ventricle, which is a widely used neuroinflammation model.

MMI-0100 or vehicle was infused into the brain by using a 32-gauge stainless steel syringe needle placed in and projecting 0.5 mm below the tip of the cannula for 2 min. This needle was connected via a PE tube to a 5-μl Hamilton syringe mounted to a Microdrive pump (KD Scientific). To allow the drug to diffuse, the infusion syringe remained in place for 1 min. For the lateral ventricle, injection volume is 2 μl, and for the bilateral hippocampus CA1, injection volume is 0.5 μl/side. After drug administration, each mouse was subjected to behavioral testing immediately. In addition, the selection of the doses was based on the previous reports and our preliminary observations (24).

For nasal application, mice were anesthetized with isoflurane and infused with MMI-0100 (25 nmol, 10 μl) using a 10-μl pipette. MMI-0100 was dissolved in 10% β-cyclodextrin for nasal application. Each of the applications to the left and right rhinarium, respectively, lasted about 1 min. Mice returned to their home cage and behavioral experiment was performed 2 h after intranasal application. To minimize non-specific stress responses during the application procedure, holding of the mice for 2–3 min was practiced for at least 3 days prior to the experiment.

The behavior experiments were carried out 15 min after i.c.v. injection of MMI-0100, or 2 h after intranasal infusion of MMI-0100. The cytokines were measured 15 min after i.c.v. injection of MMI-0100.

### Behavioral Tasks

**Figure 1:**
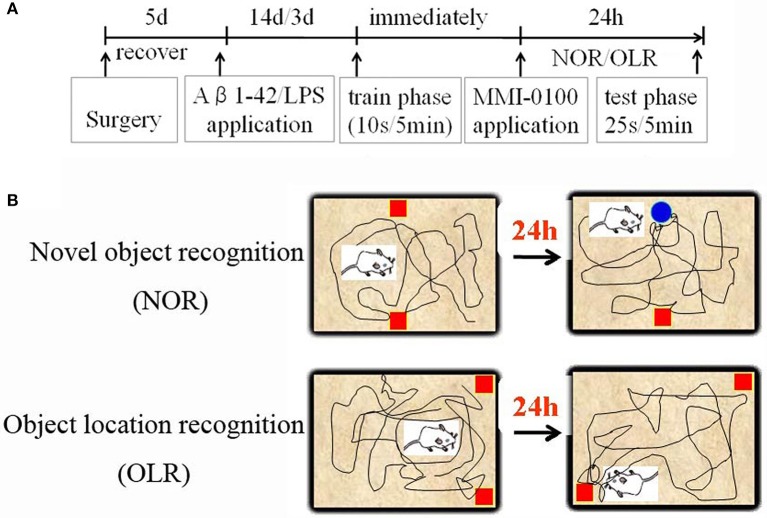
Schematic diagram for experimental design and schedule. **(A)** Animal experimental protocol indicating the time course for various interventions utilized during the experiment. **(B)** Experimental methods for NOR and OLR tasks.

The procedures for the novel object recognition (NOR) and object location recognition (OLR) tasks were described in our previous report (23, 30). Schematic diagram for experimental design and schedule were shown in Figure 1. The videos for NOR and OLR tasks were shown in Supplementary Material. Briefly, the procedure of NOR and OLR tasks consisted of two sessions: the training phase and the test phase, and the time elapsed between the training and test phases is 24 h. Each mouse was tested in its home cage in a sound-attenuated room with dim lighting. Each mouse was handled for 3 min per day for three consecutive days prior to training. In our experiments, all objects were made of plastic or glass and were similar in sizes (4–5 cm high) but different in color and shape. There were several copies of each object used interchangeably. The objects were cleaned thoroughly between trials to avoid olfactory cues. Exploration was defined as sniffing or touching the object with the nose or forepaws. Resting against or moving around the object was not considered exploratory behavior. The discrimination index (DI) in the memory retention phase was calculated as the percentage of the time a mouse explored the new object or location over the total time the mouse explored the two objects or locations. A DI of 50% is equivalent to the chance level, and a significantly higher DI indicates that the mouse had a preference for one object or location, indicating that they could remember the familiar object or location. We found no significant difference between treatments in the duration of exploration of the objects during the training phase, as well as in the duration and the total exploration time (TET) during the memory retention phase (Table 1).

**Table 1:** Duration of training phase, duration of test phase, and TET in test phase for each group.

**Figure**	**Group**	**Duration of training phase**	**Duration of test phase**	**TET in test phase**	***n***
Figure 2A	Vehicle + vehicle	173.1 ± 12.7	274 ± 17.0	16.4 ± 1.6	7
	Aβ + vehicle Aβ + MMI-0100	184.5 ± 10.2 199.4 ± 32.5	300 ± 0 300 ± 0	17.2 ± 1.5 14.1 ± 2.2	7 8
Figure 2B	Vehicle + vehicle	188.5 ± 25.4	300 ± 0	19.5 ± 1.7	7
	Aβ + vehicle Aβ + MMI-0100	195.1 ± 16.7 165.3 ± 13.6	300 ± 0 288.1 ± 13.7	20.6 ± 1.4 18.4 ± 1.2	8 8
Figures 2C,D	Vehicle + vehicle LPS + vehicle LPS + MMI-0100 Vehicle + vehicle LPS + vehicle LPS + MMI-0100	187.2 ± 17.4 199.2 ± 29.1 176.3 ± 38.8 183.4 ± 26.2 206.3 ± 20.1 226.5 ± 30.8	279.4 ± 19.8 300 ± 0 300 ± 0 300 ± 0 300 ± 0 300 ± 0	18.6 ± 2.2 13.2 ± 1.6 14.2 ± 1.4 15.1 ± 1.1 17.3 ± 1.2 13.2 ± 1.5	8 8 7 7 7 8
Figure 3A	Vehicle + vehicle Aβ + vehicle Aβ + MMI-0100	191.8 ± 24.6 217.6 ± 28.4 228.4 ± 14.1	300 ± 0 300 ± 0 278.9 ± 8.4	15.5 ± 1.5 16.9 ± 1.4 15.7 ± 1.3	7 7 7
Figure 3B	Vehicle + vehicle Aβ + vehicle Aβ + MMI-0100	236.4 ± 20.5 211.5 ± 13.1 236.3 ± 26.7	300 ± 0 288.6 ± 16.3 300 ± 0	14.2 ± 1.5 15.2 ± 0.9 19.6 ± 1.4	7 9 7
Figures 3C,D	Vehicle + vehicle LPS + vehicle LPS + MMI-0100 Vehicle + vehicle LPS + vehicle LPS + MMI-0100	197.4 ± 19.9 184.1 ± 16.4 203.7 ± 11.6 215.8 ± 17.1 195.1 ± 24.3 223.9 ± 37.2	291.6 ± 11.5 300 ± 0 300 ± 0 300 ± 0 300 ± 0 300 ± 0	15.4 ± 1.7 11.6 ± 1.7 17.1 ± 1.4 15.5 ± 1.2 14.8 ± 1.5 17.3 ± 1.3	8 7 7 9 9 9
Figure 10A	Vehicle + vehicle	213.5 ± 15.1	300 ± 0	13.3 ± 1.1	8
	Aβ + vehicle Aβ + MMI-0100	179.8 ± 27.3 198.0 ± 31.1	294 ± 1.2 300 ± 0	16.1 ± 1.3 14.1 ± 0.9	8 10
Figure 10B	Vehicle + vehicle	207.1 ± 16.3	297 ± 10.7	15.4 ± 1.1	7
	Aβ + vehicle Aβ + MMI-0100	243.8 ± 48.1 221.8 ± 24.6	300 ± 0 268.4 ± 25.4	16.3 ± 1.3 16.7 ± 1.9	7 8
Figures 10C,D	Vehicle + vehicle LPS + vehicle LPS + MMI-0100 Vehicle + vehicle LPS + vehicle LPS + MMI-0100	199.1 ± 13.7 213.2 ± 17.8 225.6 ± 31.5 178.3 ± 26.5 196.1 ± 22.4. 208.9 ± 21.8	300 ± 0 300 ± 0 300 ± 0 300 ± 0 300 ± 0 300 ± 0	17.4 ± 1.2 15.2 ± 0.9 20.3 ± 1.5 19.8 ± 1.2 17.2 ± 1.1 18.1 ± 1.7	8 10 9 9 10 8

### NOR Task

In the training phase, two identical objects were placed on opposite sides of the home cage. The training trial was ended when the mouse had explored the two identical objects for 10 s within 5 min. Animals were eliminated if TET in the training phase did not reach 10 s within 5 min. In the test phase, a novel object, and a familiar object from the training trial were placed in the same positions as in the training phase, and the test phase lasted for 5 min. The test phase was ended when the mouse had explored the two objects for a total of 25 s, or after 5 min had passed, the test phase was terminated.

### OLR Task

In the training phase, two identical objects were placed in distinct corners of the home cage. The training trial was ended when the mouse had explored the two identical objects for 10 s within 5 min. Animals were eliminated if the TET in the training phase could not reach 10 s within 5 min. In the test phase, one of the objects was moved to a new corner, which was randomly chosen, and the test phase lasted for 5 min. The test phase was ended when the mouse had explored two locations of the objects for a total of 25 s, or after 5 min had passed, the test phase was terminated.

### Quantitative PCR

Amnesia was caused by Aβ_1−42_ injection for 14 days or LPS infusion for 3 days; 15 min after injection of MMI-0100 or vehicle, the hippocampus was removed from mice. Real-time RT-PCR was performed according to a previous report (31). Total RNA was extracted using Trizol reagent (TaKaRa) and 1 μg of RNA in each sample was reversely transcribed into a single-stranded complementary DNA with the 5X PrimeScript RT Master Mix (TaKaRa) following the manufacturer's instructions. Amplification was carried out in a 25 μl reaction mixture consisting of 12.5 μl of 2X SYBR Premis Ex TaqTM II, 2 μl of cDNA, 1 μl of forward primer, 1 μl of reverse primer, and 8.5 μl of H_2_O, and was run under the following conditions: 95°C for 30 s, followed by 40 cycles of 95°C for 5 s, 58°C for 30 s, and 72°C for 30 s. The primer pair P1/P2 was used in the RT-PCR assay to identify the expression level of TNF-α, IL-6, IL-1β, and iNOS gene in vehicle + vehicle-, Aβ_1−42_ + vehicle-, Aβ_1−42_ + MMI-0100-, LPS + vehicle-, and LPS + MMI-0100-treated groups. The primers used in quantitative real-time PCR are shown in Table 2.

**Table 2:** Information on primers used in this experiment.

**Gene**	**Prime**	**Sequence (5′-3′)**
IL-1β	Sense	CAGCTTCAAATCTCGCAGCA
	Anti-sense	CTCATGTCCTCATCCTGGAAGG
TNF-α	Sense	ACTCCCAGGTTCTCTTCAAGG
	Anti-sense	GGCAGAGAGGAGGTTGACTTTC
iNOS	Sense	CGCAGCTGGGCTGTACAAAC
	Anti-sense	CTGTGGCTCCCATGTTGCATT
IL-6	Sense	ACAACCACGGCCTTCCCTA
	Anti-sense	TCATTTCCACGATTTCCCAGA
GAPDH	Sense	GCCACAGACGTCACTTTCCTAC
	Anti-sense	CGGGAACACAGTCACATACCA

*TNF-α, tumor necrosis factor-α; IL-6, interleukin-6; IL-1β, interleukin-1β; iNOS, nitric oxide synthase*.

### Enzyme-Linked Immunoassay (ELISA)

Total proteins from the hippocampus of mice in each group were extracted with RIPA lysis buffer containing protease inhibitor (Gibco, Thermo Fisher Scientific, Inc.). Total proteins were determined using a bicinchoninic acid protein assay kit (Sangon Biotech Co., Ltd.). IL-1β, IL-6, and TNF-α in the hippocampus tissue were measured using ELISA kits (cat number: E-EL-M0049 for TNF-α, cat number: E-EL-M0044 for IL-6, cat number: E-EL-M0037 for IL-1β, Elabscience), according to the manufacturer's instructions.

### Western Blotting

Western blotting was carried out as per the manufacturer's instructions (Bio-Rad Laboratories, Inc). Protein was extracted with RIPA buffer containing protease inhibitor (Gibco; Thermo Fisher Scientific, Inc.). The protein concentration was determined using a BCA protein assay kit (Pierce; Thermo Fisher Scientific, Inc.). Briefly, the protein samples (40 μg per lane) isolated from the hippocampus were separated by SDS-PAGE and electrophoretically transferred onto polyvinylidene fluoride membranes. Membranes were blocked with 5% skimmed milk for 2 h and incubated overnight at 4°C with specific first antibodies. The first antibodies in WB assay are shown in Table 3. GAPDH was used as a loading control. Subsequently, the membranes were incubated with the corresponding secondary antibodies and the reaction was visualized with chemiluminescence reagents provided with an ECL kit (Thermo) and exposed to a film. The intensity of the blots was quantified with densitometry (Image J 1.49v; National Institutes of Health).

**Table 3:** Information on antibodies used in this experiment.

**Antibody name**	**Dilution concentration**	**Cat number**	**Company**
Anti-p-MK2 Rabbit mAb Anti-t-MK2 Rabbit mAb Anti-p-p38 Rabbit mAb	1:1,000 1:1,000 1:1,000	3700S D16188-0025 4511S	CST, USA BBI Life Science CST, USA
Anti-t-p38 Rabbit mAb	1:1,000	9212S	CST, USA
Anti-p-ERK Rabbit mAb	1:1,000	4370T	CST, USA
Anti-t-ERK Rabbit mAb Anti-p-JNK Rabbit mAb Anti-t-JNK Rabbit mAb	1:500 1:1,000 1:500	AM076-1 A688T AJ518-1	Beyotime CST, USA Beyotime
GAPDH Rabbit mAb	1:1,000	5174S	CST, USA
Second antibody	1:1,000	A0208	Beyotime

### Immunostaining

Mice were anesthetized with sodium pentobarbital and then transcardially perfused with 20 ml of PBS followed by 60 ml of 4% paraformaldehyde. The brains were removed and cut into consecutive frozen sections and then incubated overnight with CD11b antibody (cat number: CBL1512, 1:100, Chemicon Biotechnology) and anti-glial fibrillary acidic protein (GFAP) (cat number: sc-166458, 1:100, Santa Cruz Biotechnology, USA). The secondary antibody (cat number: A0521, Cy3-labeled Goat Anti-Mouse IgG, 1:200, Beyotime) was applied to the sections for 40 min at 37°C. The images were performed by a fluorescent microscope (ZEISS International, optical and optoelectronic technology, USA).

### Cell Culture

BV-2 cells were obtained from cellbank.org.cn. BV-2 cells were cultured in DMEM containing 10% fetal bovine serum at 37°C in a humidified 5% CO_2_ incubator. BV-2 cells were pretreated with LPS (1 μg) for 1 h and then incubated with MMI-0100 (10^−5^ to 10^−8^ M) for 24 h. *In vitro* experiments include cell viability assessment (MTT), real-time PCR, and Western blotting. The results of cell stability are shown in Figure S1.

### NIR Fluorescence Imaging

To confirm the penetration of the MMI-0100 in the brain tissue of mice, the fluorescent dye Cy7.5 was used. The specific synthesis method is shown in Figure 10A. NIR fluorescence imaging was performed by a whole-mouse imaging system (Imaging Station IVIS Lumina II, Caliper). Two hours after intranasal administration of Cy7.5-MMI-0100 and Cy7.5 alone into the nasal cavity, mice were sacrificed and perfused with 20 ml of PBS to remove residual blood in the brain. The brain was removed and placed into an imaging system. Images were captured by a CCD camera embedded in the imaging system and analyzed by the Lumina II Living Imaging 4 software (32).

### Confocal Microscopy

Two hours after intranasal administration of Cy7.5-MMI-0100 and Cy7.5 alone into the nasal cavity, mice were sacrificed, and perfused with 20 ml of PBS to remove residual blood followed by 60 ml of 4% paraformaldehyde. The frozen brain tissue was cut into 30-μm-thick sections using a cryotome and observed with an Olympus FluoView FV1000 confocal laser scanning microscope (32).

### Statistical Analysis

All data are presented as the mean ± SEM for two repeats twice of each experiment. Statistical analysis was conducted using SPSS 19.0 (IBM Corp.). A *t* test was used to examine whether the DI of each group was significantly different from the chance level (50%). Differences among more than two groups were assessed by one-way ANOVA, followed by Bonferroni's *post-hoc* test, and *p* < 0.05 was considered to indicate a statistically significant difference.

## Results

### Central MMI-0100 Ameliorates Object and Location Recognition Memory Impairment Induced by Aβ_1−42_ and LPS

**Figure 2:**
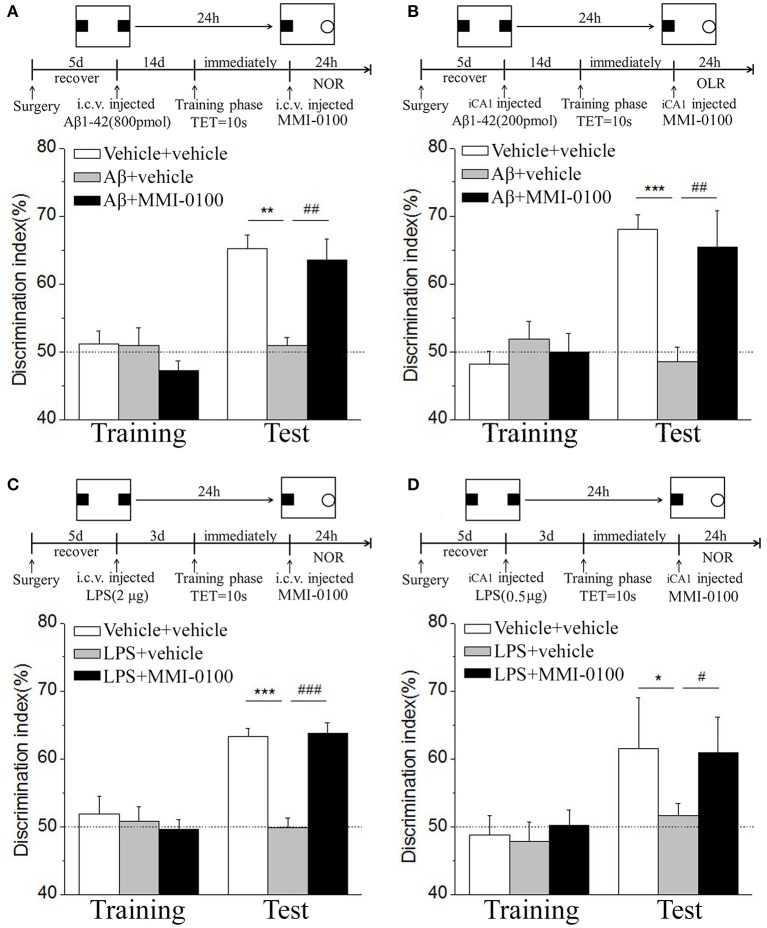
MMI-0100 attenuates Aβ_1−42_- or LPS-induced object recognition memory impairments in the NOR task. **(A,B)** Aβ_1−42_ was administered by i.c.v. or iCA1 injection at 14 days prior to MMI-0100 application. Injection of MMI-0100 (5 nmol for i.c.v., 1 nmol for iCA1) immediately after training improves amnesia elicited by Aβ_1−42_ (800 pmol for i.c.v., 200 pmol for iCA1) when tested 1 day. **(C,D)** LPS was administered by i.c.v. or iCA1 injection at 3 days prior to MMI-0100 application. Injection of MMI-0100 (5 nmol for i.c.v., 1 nmol for iCA1) immediately after training improves LPS elicited object recognition memory impairments (2 μg for i.c.v., 1 μg for iCA1) when tested 1 day. The dashed line indicates 50% chance level. *n* = 7–8 mice per group. Vertical lines represent mean ± SEM. *p < 0.05, **p < 0.01, and ***p < 0.001 compared with chance level. ^#^*p* < 0.05, ^##^*p* < 0.01, and ^###^*p* < 0.001 compared with the Aβ_1−42_ or LPS group.

**Figure 3:**
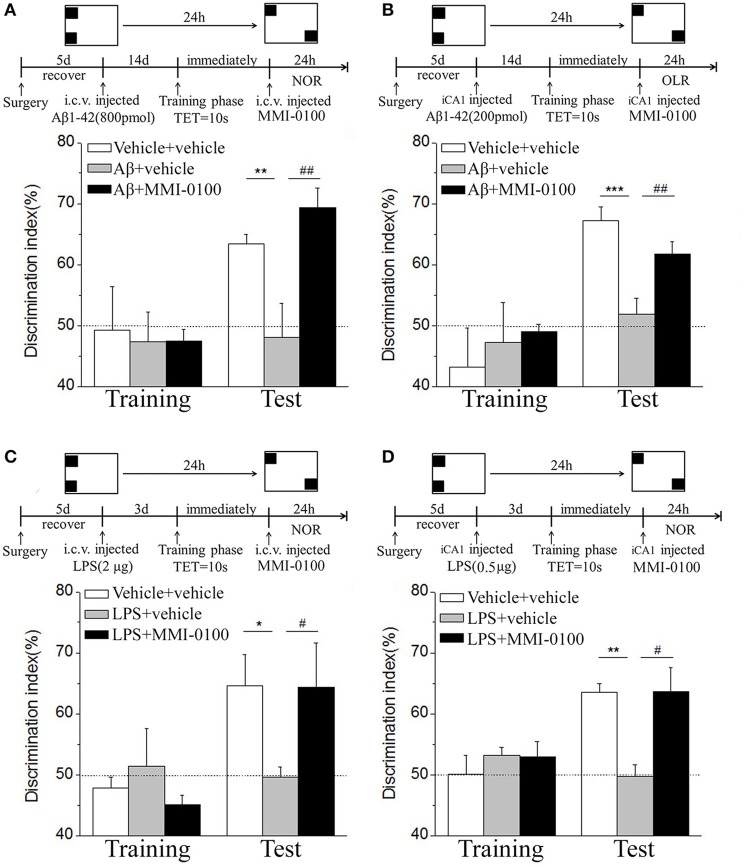
MMI-0100 ameliorates Aβ_1−42_- or LPS-induced location recognition memory impairments in OLR task. **(A,B)** In the OLR task, Aβ_1−42_ was administered by i.c.v. or iCA1 injection at 14 days prior to MMI-0100 application. Injection of MMI-0100 (5 nmol for i.c.v., 1 nmol for iCA1) immediately after training improves amnesia elicited by Aβ_1−42_ (800 pmol for i.c.v., 200 pmol for iCA1) when tested 1 day. **(C,D)** LPS was administered by i.c.v. or iCA1 injection at 3 days prior to MMI-0100 treatment. Injection of MMI-0100 (5 nmol for i.c.v., 1 nmol for iCA1) immediately after training improves LPS (2 μg for i.c.v., 1 μg for iCA1) elicited object recognition memory impairments when tested 1 day. *n* = 7–9 mice per group. Vertical lines represent mean ± SEM. The dashed line indicates 50% chance level. *p < 0.05, **p < 0.01, and ***p < 0.001 compared with chance level. ^#^*p* < 0.05 and ^##^*p* < 0.01 compared with the Aβ_1−42_ or LPS group.

In the NOR and OLR task, when the TET was 10 s and memory was tested after 24 h, vehicle + vehicle-treated mice could discriminate the novel object or location from the familiar one, with the DI (65.15% for NOR, 63.45% for OLR) being significantly higher than the 50% chance level (*p* < 0.01), but Aβ_1−42_ (i.c.v., 800 pmol) or LPS (i.c.v., 2 μg) treatment significantly disrupted memory; the average DI of this group (Aβ_1−42_: 50.96%, LPS: 49.82% for NOR; Aβ_1−42_: 48.04%, LPS: 49.62% for OLR) was significantly lower than that of vehicle + vehicle (^**^p < 0.01 for Figure 2A, ^***^p < 0.001 for Figure 2C, ^**^p < 0.01 for Figure 3A and ^*^p < 0.05 for Figure 3C). Injection of MMI-0100 (i.c.v., 5 nmol) into the lateral ventricle 14 days after injection of Aβ_1−42_ or 3 days' injection of LPS showed a good memory performance (^##^*p* < 0.01 for Figure 2A, ^###^*p* < 0.001 for Figure 2C, ^##^*p* < 0.01 for Figure 3A, and ^#^*p* < 0.05 for Figure 3C), suggesting that MMI-0100 alleviates the memory-impairing effects of Aβ_1−42_ or LPS.

In the NOR and OLR tasks, when the TET was 10 s and memory was tested after 24 h, MMI-0100 (1 nmol) infused into the bilateral hippocampus displayed a good memory performance, compared to the Aβ_1−42_ (200 pmol) or LPS (1 μg) group (^***^p < 0.001 for Figure 2B, ^*^p < 0.05 for Figure 2D, ^***^p < 0.001 for Figure 3B, and ^**^p < 0.01 for Figure 3D; ^##^*p* < 0.01 for Figure 2B, ^#^*p* < 0.05 for Figure 2D, ^##^*p* < 0.01 for Figure 3B, and ^#^*p* < 0.05 for Figure 3D).

### MMI-0100 Suppresses Microglia and Astrocyte Activation and Reduces Inflammatory Factors

**Figure 4:**
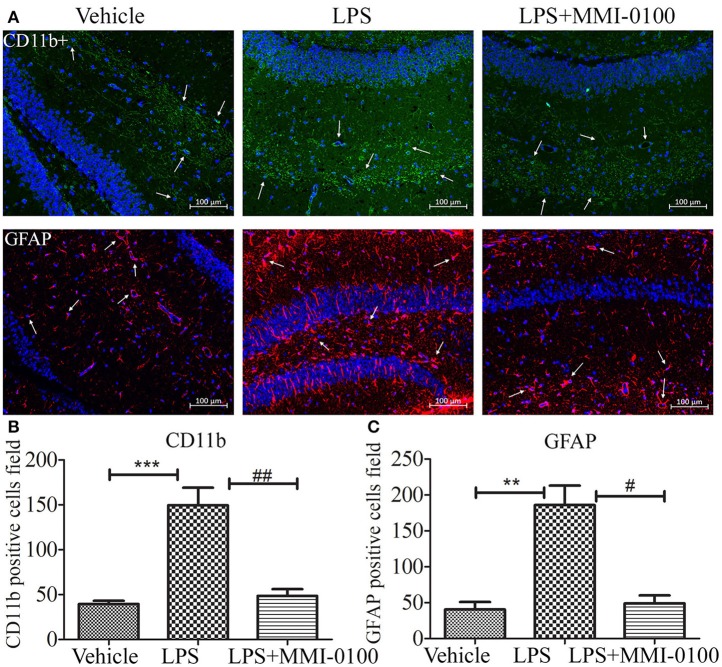
Effects of MMI-0100 treatment in LPS induced the activation of microglia and astrocytes. **(A)** Representative confocal images of mice administered with the vehicle saline, LPS, or LPS + MMI-0100 are shown. Hippocampal histology sections were double-labeled for CD11b+ (green) and DAPI (blue) or GFAP (red) and DAPI (blue), and assessments were made with immunofluorescence microscopy, scale bars = 100 μm, 200×. **(B,C)** The quantification of GFAP-positive astrocytes and CD11b-positive microglia is described. Data are mean ± SEM (*n* = 5 mice per group). Data were analyzed using one-way ANOVA followed by Bonferroni's *post-hoc* test. **p < 0.01 and ***p < 0.001 compared with control. ^#^*p* < 0.05 and ^##^*p* < 0.01 compared with the LPS group.

**Figure 5:**
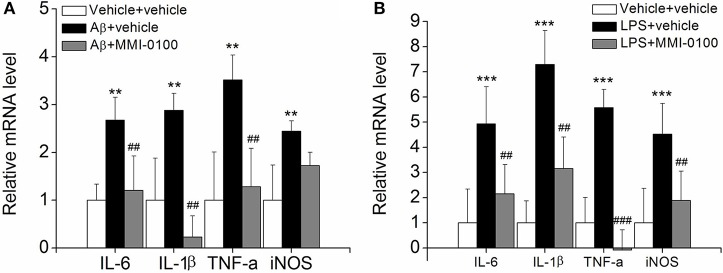
Effects of MMI-0100 treatment on the mRNA level of pro-inflammatory factors in the hippocampus. **(A,B)** The mRNA levels of pro-inflammatory cytokines, IL-6, IL-1β, TNF-α, and iNOS were determined by real-time PCR. Note that IL-6, IL-1β, TNF-α, and iNOS levels were significantly increased in the Aβ_1−42_ group (*n* = 5) or LPS group (*n* = 5), and this increase was counteracted by treatment with MMI-0100 (*n* = 5). Vertical lines represent mean ± SEM. **p < 0.01 and ***p < 0.001 compared with vehicle + vehicle. ^##^*p* < 0.01 and ^###^*p* < 0.001 compared with the Aβ_1−42_ or LPS group.

**Figure 6:**
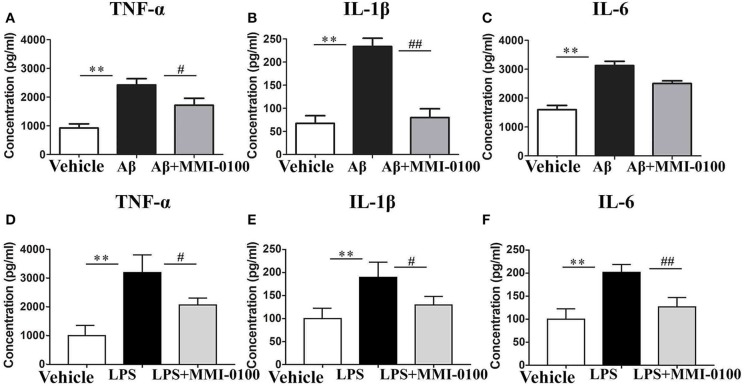
Effects of MMI-0100 treatment on the protein expression level of pro-inflammatory factors in the hippocampus. Hippocampal levels of pro-inflammatory cytokines, TNF-α **(A,D)**, IL-1β **(B,E)**, and IL-6 **(C,F)** were evaluated by ELISA assay. Note that TNF-α, IL-1β, and IL-6 levels showed significant increase in the Aβ_1−42_ group or LPS group, and following MMI-0100 treatment, the Aβ_1−42_ + MMI-0100 group recovered to control levels. *n* = 5 mice per group. Vertical lines represent mean ± SEM. **p < 0.01 compared with the vehicle group vs. the Aβ_1−42_ or LPS group. ^#^*p* < 0.05 and ^##^*p* < 0.01 compared with the Aβ_1−42_ or LPS group vs. the Aβ_1−42_ + MMI-0100 or LPS + MMI-0100 group.

To determine the effect of MMI-0100 on glial activation, immunofluorescent staining for GFAP and CD11b in the hippocampi of mice was performed. GFAP and CD11b are markers of astrocyte and microglia. As shown in Figure 3, LPS increased CD11b and GFAP expression, while MMI-0100 treatment lowered the expression of both markers, suggesting that MMI-0100 inhibited the activation of microglia and astrocytes induced by LPS in mice (Figure 4). In addition, a series of pro-inflammatory cytokines, such as IL-6, IL-1β, TNF-α, and iNOS, were dramatically increased compared with control in the hippocampus of LPS- or Aβ_1−42_-induced AD model mice (*p* < 0.01). However, these pro-inflammatory factors were significantly down-regulated after MMI-0100 application (*p* < 0.01, Figures 5A,B). Furthermore, the protein levels of IL-6, IL-1β, and TNF-α were higher in the hippocampus of LPS- or Aβ_1−42_-induced AD model mice, and MMI-0100 treatment markedly down-regulated the expression of IL-6, IL-1β, and TNF-α (Figure 6). Overall statistical comparisons by one-way ANOVA followed by Bonferroni's *post-hoc* tests and these results indicated that MMI-0100 inhibited the microglia activation and reduced the inflammatory factors in LPS- or Aβ_1−42_-induced AD model mice.

### MMI-0100 Down-Regulates the Phosphorylation of the MK2 Pathway and Anti-inflammatory Effect Is Not Mediated via MAPK

**Figure 7:**
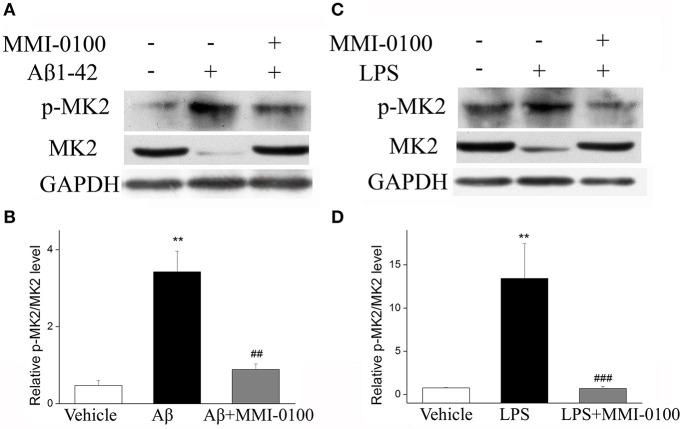
Effects of MMI-0100 treatment on the expression of pMK2 in the hippocampus. **(A,C)** Western blot analysis of pMK2 in the hippocampus in each group. **(B,D)** B/D is quantification of pMK2 expressed as the ratio of pMK2/tMK2. Values were expressed as mean ± SEM, *n* = 5 in each group. **p < 0.01 compared with the vehicle group vs. the Aβ_1−42_ or LPS group, ^##^*p* < 0.01 and ^###^*p* < 0.001 compared with the Aβ_1−42_ or LPS group vs. Aβ_1−42_ + MMI-0100 or LPS + MMI-0100 group.

**Figure 8:**
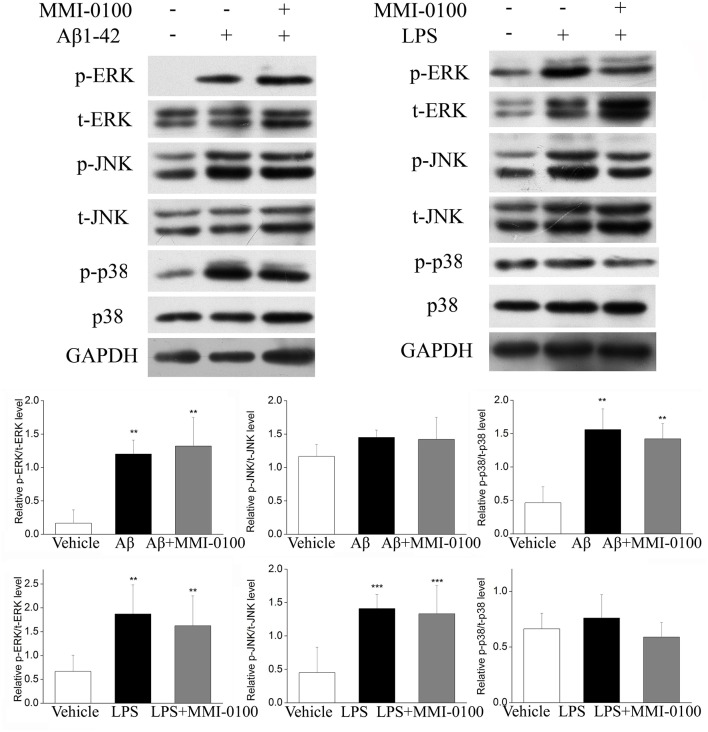
Effect of MMI-0100 on Aβ_1−42_- or LPS-induced activation of MAPK in the hippocampus in mice. The upper panel is a representative immunoblot of pERK, tERK, pJNK, tJNK, p38, and p-p38 detected by Western blotting; the lower panel is quantification of MAPK signaling pathway-related protein expressed as the ratio of pERK/tERK, pJNK/tJNK, and p-p38/p38. Values were expressed as mean ± SEM, *n* = 5 in each group. **p < 0.01 and ***p < 0.001 compared with vehicle group.

To determine the mechanism of MMI-0100 in suppression of neuroinflammation, we further investigated the expression of MK2 signaling pathways and MAPK signaling pathways by Western blotting. As shown in Figure 7, the phosphorylation of MK2 was significantly up-regulated in the hippocampus of LPS or Aβ_1−42_-induced AD model mice, while MMI-0100 treatment markedly decreased p-MK2. However, in Figure 8, i.c.v. administration of MMI-0100 could not decrease the phosphorylation of ERK, JNK, and p38 in the hippocampus of LPS- or Aβ_1−42_-induced AD model mice.

### MMI-0100 Has an Anti-inflammatory Effect in BV-2 Microglial Cells

**Figure 9:**
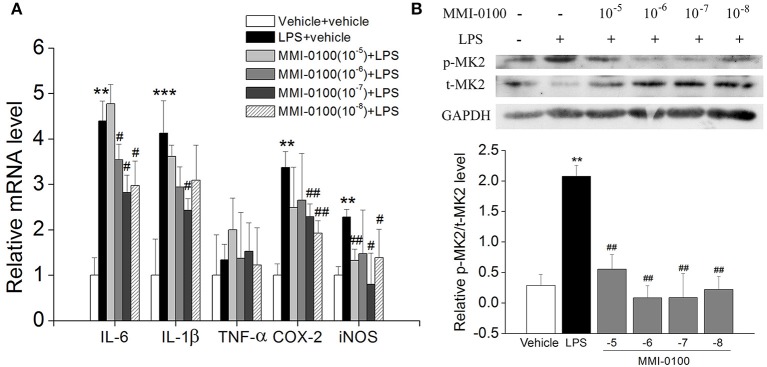
Effects of MMI-0100 treatment on LPS-induced inflammatory response and the expression of pMK2 in BV-2 cells. **(A)** MMI-0100 reduces LPS (1 μg/ml)-induced release of pro-inflammatory mediators such as IL-6, IL-1β, TNF-α, COX-2, and iNOS level in BV-2 cells. **(B)** The upper panel is a representative immunoblot of pMK2, tMK2, and GAPDH detected by Western blotting; the lower panel is quantification of MK2 expressed as the ratio of pMK2/tMK2. **p < 0.01 compared with vehicle group vs LPS group. ^#^*p* < 0.05 and ^*##*^*p* < 0.01 compared with the LPS group vs. LPS + MMI-0100 (10^−5^, 10^−6^, 10^−7^, and 10^−8^ M) at different doses. Values were expressed as mean ± SEM.

Subsequently, we confirmed the anti-inflammatory response of MMI-0100 in LPS-treated BV-2 cells. As shown in Figures S1A,B, we investigated the most suitable dose of LPS to stimulate BV2 cells by MTT assay. Similar to the results *in vivo*, the levels of IL-6, IL-1β, COX-2, and iNOS were dramatically increased in the BV2 cells after LPS treatment (*p* < 0.01, Figure 9A). MMI-0100 treatment significantly decreased expression of IL-1β, IL-6, COX-2, and iNOS (*p* < 0.05, Figure 9A). As shown in Figure 9B, the phosphorylation of MK2 was significantly up-regulated in the BV2 cells, while MMI-0100 treatment markedly decreased p-MK2. These results are consistent with the results *in vivo*. In addition to BV2 cells, we similarly investigated the anti-inflammatory effects of MMI-0100 in HT22 (mouse hippocampal neuronal cell line), SH-SY5Y (a human derived neuroblastoma cell line), and U251 (glioma cell lines) cells *in vitro*. The results showed that MMI-0100 can exhibit anti-inflammatory effects on these four different cells. The detail results are shown in Figures S2–S4.

### Intranasal Administration of MMI-0100 Ameliorates Object and Location Recognition Memory Impairment Induced by Aβ_1−42_ and LPS

**Figure 10:**
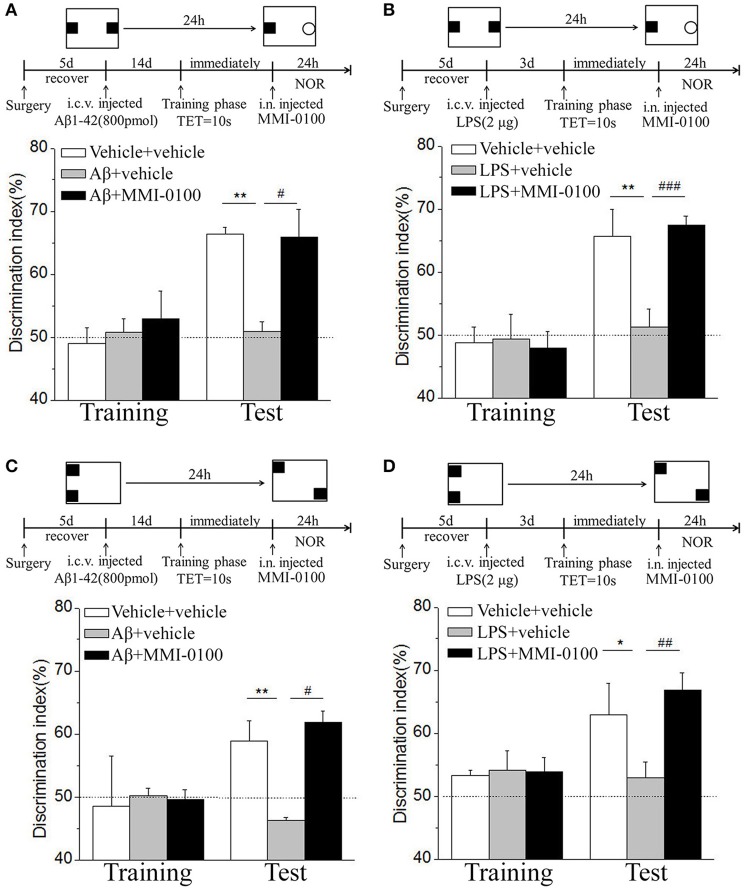
Intranasal administration of MMI-0100 ameliorates novel object and location recognition memory impairment induced by Aβ_1−42_ and LPS. **(A,B)** In the NOR task, intranasal administration of MMI-0100 (25 nmol) improves object recognition memory impairment elicited by Aβ_1−42_ (800 pmol for i.c.v.) injected 14 days or LPS (2 μg) injected 3 days before training when tested 1 day. **(C,D)** In the OLR task, intranasal administration of MMI-0100 (25 nmol) improves object location recognition memory impairment elicited by Aβ_1−42_ (800 pmol, i.c.v.) injected 14 days or LPS (2 μg, i.c.v.) injected 3 days before training when tested 1 day. The dashed line indicates 50% chance level, *p < 0.05 and **p < 0.01 compared with chance level. ^#^*p* < 0.05, ^##^*p* < 0.01, and ^###^*p* < 0.001 compared with the Aβ_1−42_ or LPS group. *n* = 8–10 mice per group. Vertical lines represent mean ± SEM.

In the NOR and OLR tasks, when the TET was 10 s and memory was tested after 24 h, vehicle + vehicle-treated mice could discriminate the novel object or location from the familiar one, with the DI (66.32% for NOR, 58.96% for OLR) being significantly higher than the 50% chance level (*p* < 0.01), Aβ_1−42_ (i.c.v., 800 pmol) or LPS (i.c.v., 2 μg) treatment significantly disrupted memory, and the average DI of this group (Aβ_1−42_: 50.98%, LPS: 51.35% for NOR; Aβ_1−42_: 46.31%, LPS: 52.97% for OLR) was significantly lower than that of vehicle + vehicle. Intranasal administration of MMI-0100 (25 nmol), 14 days after injection of Aβ_1−42_ or 3 days after injection of LPS, showed a good memory performance (^**^p < 0.01 for Figure 10A, ^**^p < 0.01 for Figure 10B, ^**^p < 0.01 for Figure 10C, and ^*^p < 0.05 for Figure 10D; ^#^*p* < 0.05 for Figure 10A, ^###^*p* < 0.001 for Figure 10B, ^#^*p* < 0.05 for Figure 10C, and ^##^*p* < 0.01 for Figure 10D).

### Near-Infrared Fluorescent and Confocal Microscopy Experiments Were Applied to Detect the Brain Uptake and Distribution of Intranasal MMI-0100

**Figure 11:**
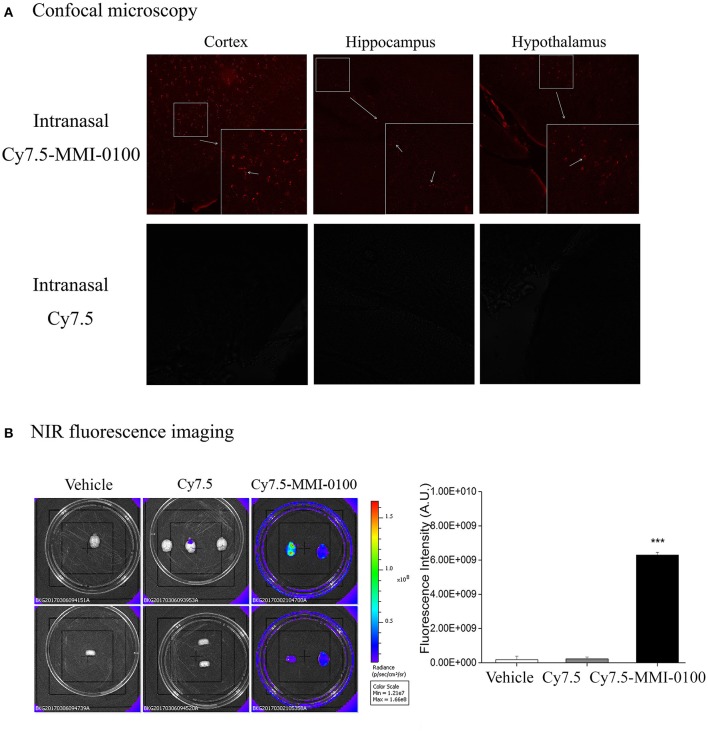
**(A)** Confocal microscopy images of cortex, hippocampus, and hypothalamus of mouse brain after intranasal injection with Cy7.5 alone or Cy7.5-MMI-0100. **(B)** *Ex vivo* NIR fluorescence imaging and fluorescence intensity of perfused mouse brains 2 h after intranasal injection of the Cy7.5-MMI-0100, Cy7.5 alone, and saline as controls. All data are expressed as mean ± SEM and were analyzed by one-way ANOVA followed by Dunnett's *post-hoc* test. *n* = 4/group. ***p < 0.001 compared with Cy7.5 vs. Cy7.5-MMI-0100. Vertical lines represent mean ± SEM.

To demonstrate the delivery of MMI-0100 into the mouse brain, *in vivo* fluorescence imaging experiments were performed. Mice were killed and perfused for removal of the residual blood in brain, and the brains were dissected for ex vivo fluorescence imaging. Cy7.5-MMI-0100 was distributed to the whole brain immediately 2 h after intranasal injection (Figure 11). Figure 11 shows the real-time *in vivo* bio-distribution for Cy7.5-MMI-0100. Brains from control mice treated with Cy7.5 or saline alone displayed no significant signal.

## Discussion

Targeting of these immune mechanisms could lead to future therapeutic or preventive strategies for AD (6). The p38 MAPK signaling pathways play a key role in neuroinflammation, and the anti-neuroinflammatory effect of the p38 MAPK inhibitor MW01-2-069A-SRM is associated with decreased phosphorylation state of MK2 in preclinical and clinical trials (10). Although targeting p38 MAPK is a promising option for the treatment of neural diseases, side effects of p38 inhibitors including hepatotoxicity, cardiotoxicity, and undisclosed CNS toxicity are unacceptable safety profile for drug development (9). MK2 is downstream kinase of p38 MAPK, and suppression of MK2 activity also leads to down-regulation of inflammatory cytokine expression such as TNF-α, IL-1β, and IL-6 (13, 14, 33). Thus, MK2 was unveiled as a promising option for the treatment of neural diseases.

MMI-0100, a cell-permeable peptide inhibitor of MK2, exhibits anti-inflammatory effects, including reduction of fibrosis, apoptosis, and systolic dysfunction, and is currently in clinical trials for the treatment of pulmonary fibrosis (17, 34, 35). In our study, i.c.v. administration of Aβ_1−42_ or LPS impaired memory functions in mice, which is consistent with previous reports (36, 37). The present study, for the first time, demonstrated that i.c.v. administration of MMI-0100, a peptide consisting 22 amino acids, could significantly ameliorate the memory deficit induced by Aβ_1−42_ or LPS in object and location memory tasks. Multiple reports indicate that the hippocampus plays an important role in cognition, and its dysfunction could damage the procession of memory (38, 39). Further investigation shows that intrahippocampus MMI-0100 alleviates the memory-impairing effects of Aβ_1−42_ or LPS.

The underlying mechanism of this neuroprotection of MMI-0100 may be involved in inhibiting the activation of glial cells and production of pro-inflammatory cytokines. In this study, i.c.v. administration of LPS significantly increased markers of microglia (CD11b) and astrocytes (GFAP) expression, while the expression of both markers was markedly decreased in the MMI-0100-treated mice. These results suggest that MMI-0100 suppressed the activation of microglia and astrocytes induced by LPS in mouse brain. Meanwhile, qRT-PCR and Western blotting experiments were applied to detect that a series of pro-inflammatory cytokines, including IL-6, IL-1β, TNF-α, and iNOS, were dramatically increased in the hippocampus of LPS- or Aβ_1−42_-treated mice. Furthermore, these pro-inflammatory factors were significantly down-regulated after MMI-0100 application. Taken together, these results show that MMI-0100 ameliorates the memory deficit induced by Aβ_1−42_ or LPS in object and location memory tasks, possibly via inhibiting the microglia, and astrocyte activation and reduced inflammatory processes. Subsequently, our data further showed that the phosphorylation of MK2 was significantly up-regulated in the hippocampus of LPS- or Aβ_1−42_-induced AD model mice, which is in line with previous works (12, 40). MMI-0100 treatment markedly inhibited phosphorylation of MK2, but not the phosphorylation of ERK, JNK, and p38 by Western blotting. This study demonstrated that MMI-0100 significantly inhibited inflammatory response by attenuating LPS- or Aβ_1−42_-induced MK2 phosphorylation *in vivo*.

Microglia, the macrophages and immune defense of the brain in CNS, are activated to secrete several pro-inflammatory cytokines and neurotoxic mediators around the amyloid plaques in AD brain (4, 6). We therefore were interested in the anti-inflammatory effects of MMI-0100 in LPS-treated BV-2 microglia cells. Indeed, the results demonstrated a reduction in the expression of the pro-inflammatory mediators such as IL-6, IL-1β, TNF-α, COX-2, and iNOS upon co-treatment with MMI-0100 in LPS-treated BV-2 microglia cells. Meanwhile, the phosphorylation of MK2 could be reduced dramatically with MMI-0100. Overall, our findings suggested that MMI-0100 inhibited LPS-induced neuroinflammation through the MK2 pathway, indicating that MMI-0100 could be a potential drug for the treatment of AD.

Intranasal infusion allows drugs to rapidly enter the CNS via intracellular neuronal olfactory and extracellular trigeminal associated pathways, bypassing the BBB to effectively interact with their receptors in multiple brain regions (19, 20, 41). Due to the failure of Solanezumab in Phase III trials, brain-targeting drugs that can cross the BBB is the most important consideration (18). Given that MMI-0100 is a cell-permeant MK2 inhibitor peptide, our work also underscores the clinical potential of intranasal MMI-0100 treatment. The results showed that intranasal administration of MMI-0100 significantly ameliorated the memory deficit induced by LPS or Aβ_1−42_ in NOR and OLR tasks. To demonstrate the delivery of MMI-0100 into the mouse brain, ex vivo fluorescence imaging experiments were performed. A strong NIR fluorescent signal coming from Cy7.5-MMI-0100 was observed over the whole brain at 2 h after intranasal injected peptide. Brains from control mice treated with Cy7.5 or saline alone displayed no significant signal. Intranasal MMI-0100 ameliorates the memory deficit induced by LPS or Aβ_1−42_, and this route of administration has the potential for non-invasive therapeutic intervention for depression.

## Conclusions

Overall, the present study firstly demonstrated that central injection of MMI-0100 relieves memory impairment induced by LPS or Aβ_1−42_ and provides neuroprotection against LPS- or Aβ_1−42_-induced neuroinflammation *in vitro* and *in vivo*, and the mechanisms may be related with down-regulation of MK2 pathways. Meanwhile, intranasal administration of MMI-0100 significantly ameliorates the memory deficit induced by Aβ_1−42_ or LPS in object and location memory tasks. Near-infrared fluorescent and confocal microscopy experiments were applied to detect the brain uptake and distribution of intranasal MMI-0100. Together, the previous reports and present results suggest that MK2 protein target may represent a novel treatment strategy for neuroinflammation disease. Moreover, intranasal infusion of MMI-0100 bypassed the BBB to rapidly enter the brain, which is a novel promising treatment for the CNS disease.

## Data Availability Statement

The datasets used and/or analyzed during the current study are available from the corresponding author on reasonable request.

## Ethics Statement

All of the procedures in this experiment were approved by the Ethics Committee of Lanzhou University, China (Permit No: SYXK Gan 2009–0005).

## Author Contributions

JJ, ZW, XL, and XC conducted the experiments, performed the analysis, and wrote the manuscript. YN analyzed the data and imaging. YN and XC facilitated the equipment and software to perform the behavior experiments. SL and HX provided peptide drug MMI-0100. CM designed the experiments and contributed to writing and editing the manuscript. All authors read and approved the final manuscript.

### Conflict of Interest

The authors declare that the research was conducted in the absence of any commercial or financial relationships that could be construed as a potential conflict of interest.

## Supplementary Material

Supplementary Videos 1 and 2 NOR and OLR tasks.

## Abbreviations

NOR: novel object recognition task
OLR: object location recognition task
IL-1β: interleukin 1 beta
IL-6: interleukin-6
iNOS: inducible nitric oxide synthase
LPS: lipopolysaccharide
TNF-α: tumor necrosis factor alpha
COX-2: cyclooxygenase-2
AD: Alzheimer's disease
MK2: mitogen-activated protein kinase activated protein kinase II
CNS: central nervous system
IN: intranasal
i.c.v.: intracerebroventricular
H&E: hematoxylin and eosin stain
DMSO: dimethyl sulfoxide
DI: discrimination index
TET: total exploration time.
