# Supplementary material for: Intranasal MMI-0100 Attenuates Aβ1−42- and LPS-Induced Neuroinflammation and Memory Impairments via the MK2 Signaling Pathway
Source: Front Immunol. 2019 Nov 26;10:2707. doi: 10.3389/fimmu.2019.02707 (PMC6901946; doi:10.3389/fimmu.2019.02707)

Supplementary material

**Fig S1:** Cell viability of LPS-treated BV-2 cells was analyzed using MTT assay.

**
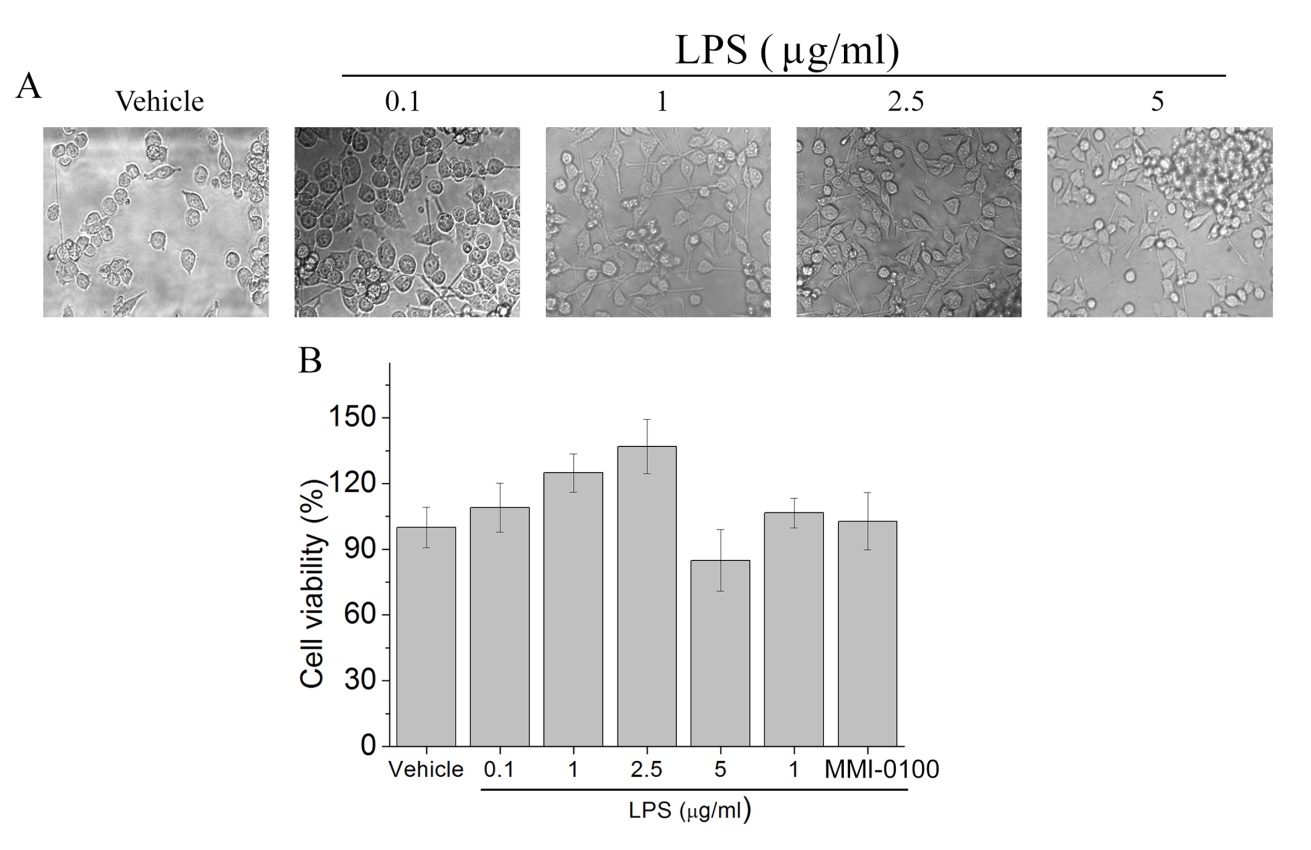
**

**Fig S2:** MMI-0100 reduces LPS (1 μg/ml)-induced release of pro-inflammatory mediators in HT22 cells.


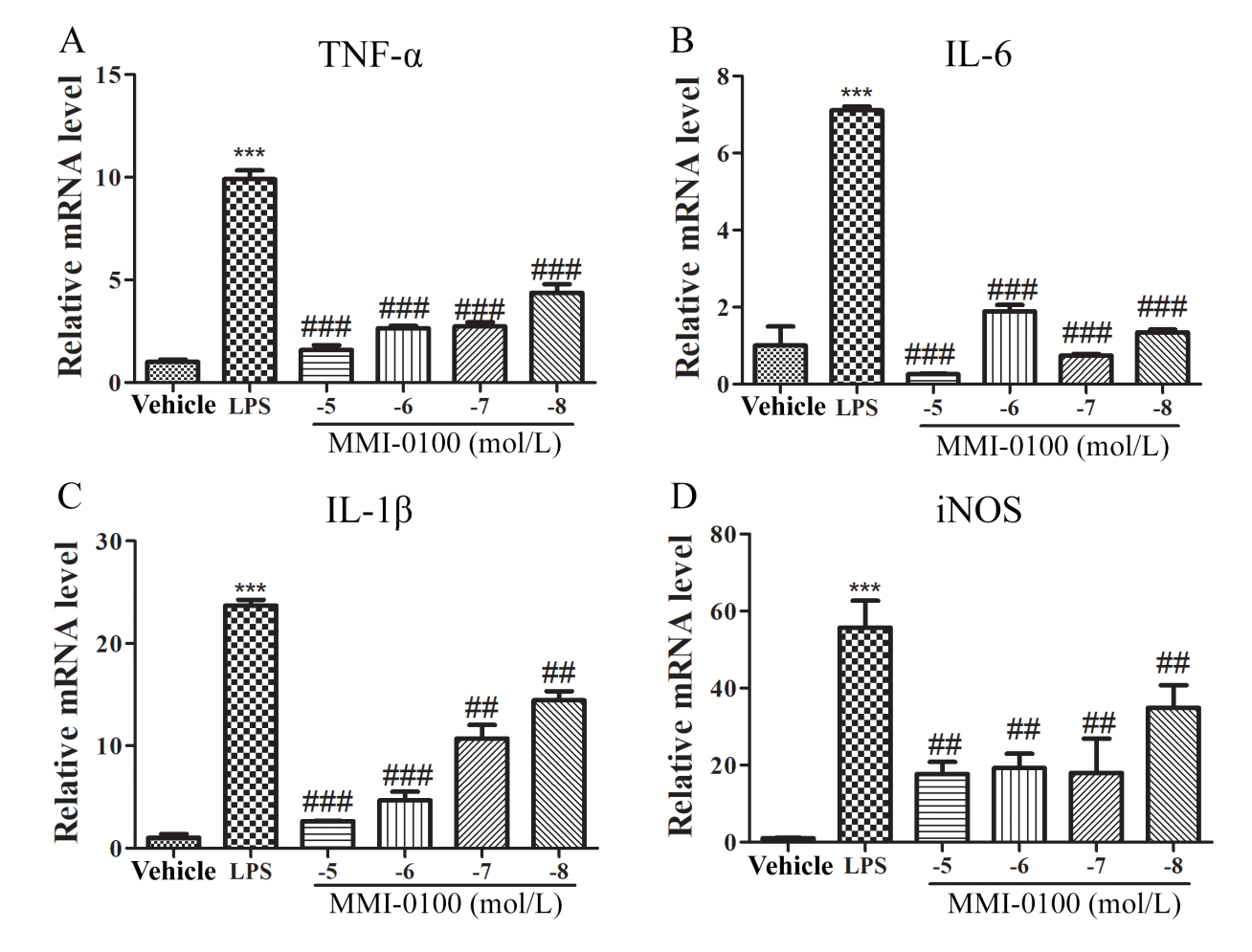


**Fig S3:** MMI-0100 reduces LPS (1 μg/ml)-induced release of pro-inflammatory mediators in SH-SY5Y cells.


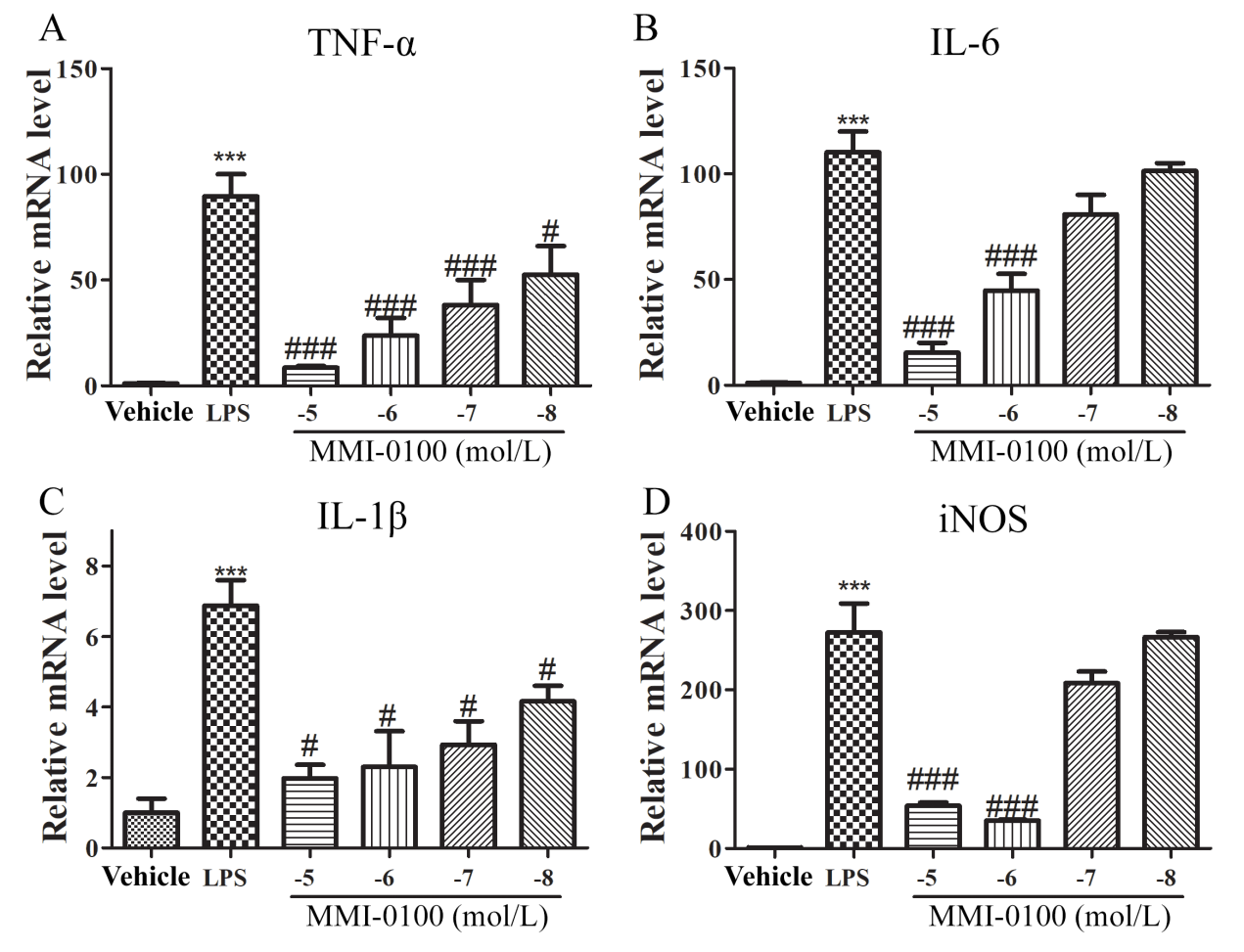


**Fig S4:** MMI-0100 reduces LPS (1 μg/ml)-induced release of pro-inflammatory mediators in U251 cells.


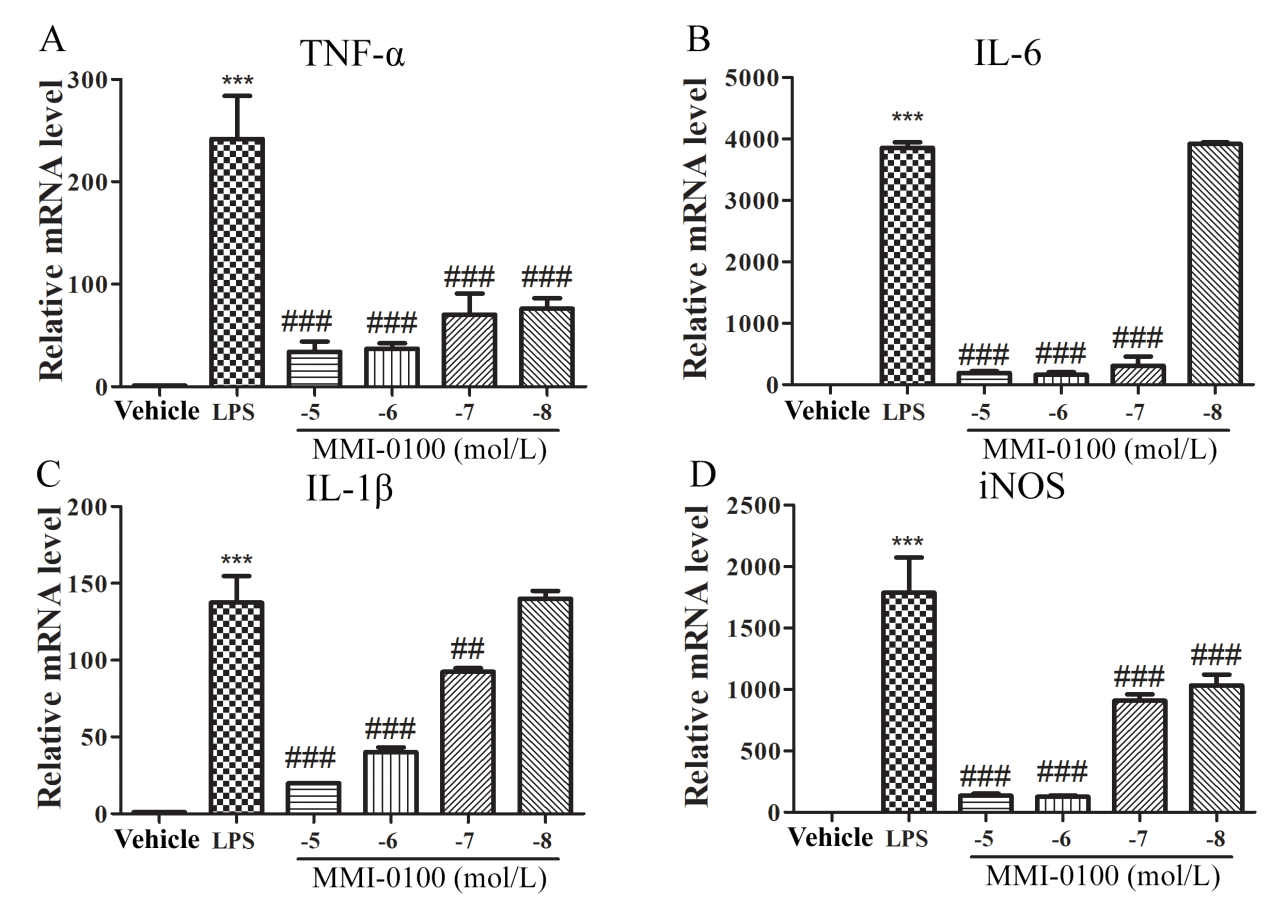


**Fig S5:** Stability of MMI-0100 in mouse brain homogenates. The peptide levels after incubation were expressed as percent parent remaining. Points indicate the means and vertical lines indicate the SEM of 3 separate experiments.


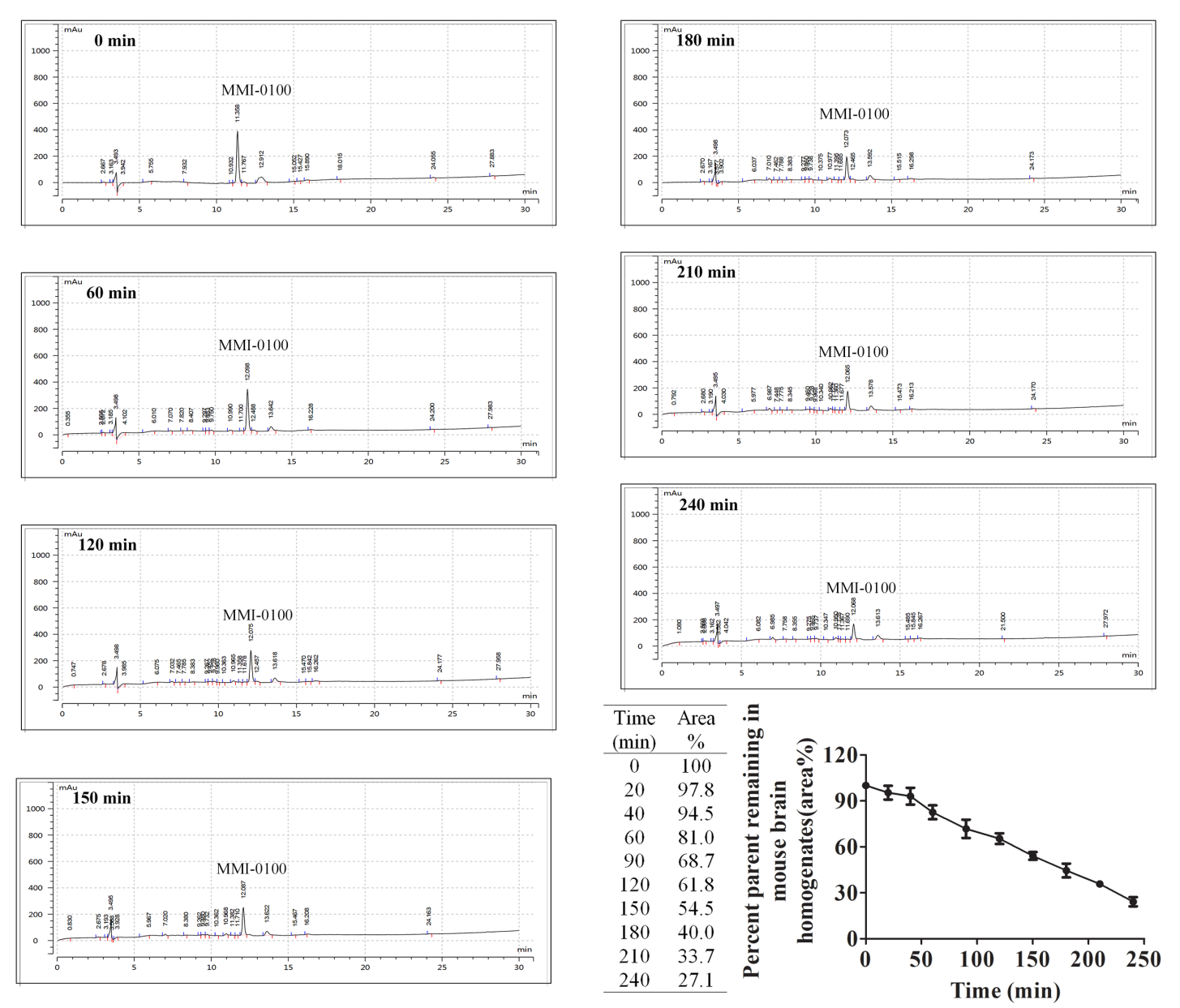

Supplement: Supplementary file 1 [file Table_1.DOCX]
